# Sirevirus LTR retrotransposons: phylogenetic misconceptions in the plant world

**DOI:** 10.1186/1759-8753-4-9

**Published:** 2013-03-04

**Authors:** Alexandros Bousios, Nikos Darzentas

**Affiliations:** 1 Institute of Applied Biosciences, Centre for Research and Technology Hellas, Thessaloniki 57001, Greece; 2 Central European Institute of Technology, Masaryk University, Brno, Czech Republic

**Keywords:** Sirevirus, Transposable elements, LTR retrotransposons, ICTV, Phylogenetic analysis, Pseudoviridae, Copia, Plant genomes, Maize

## Abstract

Sireviruses are an ancient and plant-specific LTR retrotransposon genus. They possess a unique genome structure that is characterized by a plethora of highly conserved sequence motifs in key domains of the non-coding genome, and often, by the presence of an envelope-like gene. Recently, their crucial role in the organization of the maize genome, where Sireviruses occupy approximately 21% of its nuclear content, was revealed, followed by an analysis of their distribution across the plant kingdom. It is now suggested that Sireviruses have been a major mediator of the evolution of many plant genomes. However, the name ‘Sirevirus’ has caused confusion in the scientific community in regards to their classification within the LTR retrotransposon order and their relationship with viruses - a situation that is not unique to Sireviruses, but also affects other LTR retrotransposon genera. Here, we clarify the phylogenetic position of Sireviruses as typical LTR retrotransposons of the *Copia* superfamily and explain that the confusion stems from the discrepancy in the categorization of LTR retrotransposons by the two main classification systems: the International Committee on the Taxonomy of Viruses (ICTV) system and the unified classification system for eukaryotic transposable elements. While the name ‘Sirevirus’ has been given by ICTV, we show that the transposable element system, which is more suitable for eukaryotic genome studies, lacks an appropriate taxonomic level for describing them. We urge for this inconsistency to be addressed. Finally, we provide data suggesting that of the three ICTV-proposed genera of the *Pseudoviridae* (that is, *Copia*) family, only Sireviruses form a monophyletic group, while the phylogenetic distinction between Pseudoviruses and Hemiviruses is unclear. We conclude that because of their ongoing important contribution to the classification of transposable elements, these schemes need to be frequently revisited and revised - as shown by the example of the Sirevirus LTR retrotransposon genus.

## Background

There are two main classification systems that include LTR retrotransposons (LTR-RTNs) in their taxonomies: (1) the International Committee on the Taxonomy of Viruses (ICTV), which categorizes the plethora of viruses into a single scheme that reflects their evolutionary relationships [1]; and (2) the unified classification system for eukaryotic transposable elements (TEs), which was proposed in a seminal 2007 *Nature Review* paper [2], and provides standardized nomenclature rules and simple classification strategies for the efficient identification of eukaryotic TEs - these include the 80-80-80 rule, which allocates in the same family TEs of minimum length of 80 bp with >80% sequence similarity in >80% of the length of their coding/internal domain, or of their terminal repeat regions, or both. Other available classification systems such as the Springer Index of Viruses [3], the ITIS Catalogue of life [4], and the Description of Plant Viruses (DPV) index [5] use the same nomenclature as ICTV (see below).

Based on structural and coding sequence similarities of their genomes, LTR-RTNs are evolutionarily related to retroviruses. The most plausible scenario suggests that retroviruses evolved from *Gypsy* LTR-RTNs after the acquisition of an envelope gene [6], which permitted an infectious extracellular stage. Due to this relationship, and although a limited number of LTR-RTNs contain a putative envelope-like gene, of which only the *Gypsy* element in *Drosophila* has been found to be infectious [7], ICTV has incorporated LTR-RTNs in its virus-based scheme and classified them as *Pseudoviridae* (that is, *Copia*) and *Metaviridae* (that is, *Gypsy*). Both families are divided in three genera: for *Pseudoviridae* these are the Sirevirus, Pseudovirus, and Hemivirus genera.

This article focuses on Sireviruses and their position within the virus- and TE-based classification systems. We highlight an inconsistency where only ICTV, the less appropriate system for classifying LTR-RTNs, includes a description for the taxonomic level that corresponds to Sireviruses. We argue that this taxonomic gap in the TE-based system should be rectified, as it currently confuses the scientific community, especially in genome annotation studies where ICTV is not broadly used. Finally, we indicate that only Sireviruses form a monophyletic group within the *Pseudoviridae* and that the phylogenetic basis for the division of Pseudoviruses and Hemiviruses is unclear.

## Discussion

### Research on Sireviruses

Sireviruses are an ancient LTR-RTN genus, and the only one that has exclusively proliferated within the plant kingdom. Their position within the evolutionary history of LTR-RTNs across the eukaryotic tree of life, and how they emerged in the flowering plant lineage, has been beautifully depicted by Llorens *et al*. [8]. It is the only *Pseudoviridae* genus whose members may contain a putative envelope-like gene [9]. Due to their host preference they were originally termed Agroviruses [10], before being renamed to Sireviruses by ICTV. Single Sirevirus elements such as *SIRE1*[11,12], *Opie*, and *Ji *[13,14] have been extensively studied; however, with the exception of *SIRE1*, the Sirevirus origin of most of these has often been neglected. There have also been a limited number of studies that have collectively analyzed Sireviruses on the genus level [9,10,15,16], or correctly annotated as such the Sirevirus part (or other ICTV-derived LTR-RTN genera) of the TE complements of sequenced genomes [17,18].

Recently, a series of publications from our group shed new light on this LTR-RTN genus and made it possible to properly uncover and discuss their integrative impact on their host genomes. Their unique structure among LTR-RTNs was initially revealed, characterized by a multitude of highly conserved sequence motifs within the extremely divergent non-coding part of their genome [19]. An algorithm for their accurate identification was then developed [20], followed by the elucidation of their crucial role in the organization and evolution of the maize genome, of which Sireviruses occupy approximately 21% and 90% of the *Copia* population [21]. Most recently, the MASiVEdb database was released that catalogues their distribution and abundance across a wide range of plant hosts [22] (http://bat.infspire.org/databases/masivedb/). Overall, it is now hypothesized that the amplification/removal cycles of Sireviruses may have been an important factor in the evolution and current make-up of many plant genomes.

### Taxonomic inconsistencies and classification suggestions

There exists uncertainty in the scientific community, in particular non-virologist experts, in regards to the names of the ICTV-derived LTR-RTN genera. Possibly due to their ‘virus’ suffix, scientists are misled to believe that Sireviruses (and the other *Pseudoviridae* and *Metaviridae* genera) are viral species and not typical LTR-RTNs.

**Figure 1:**
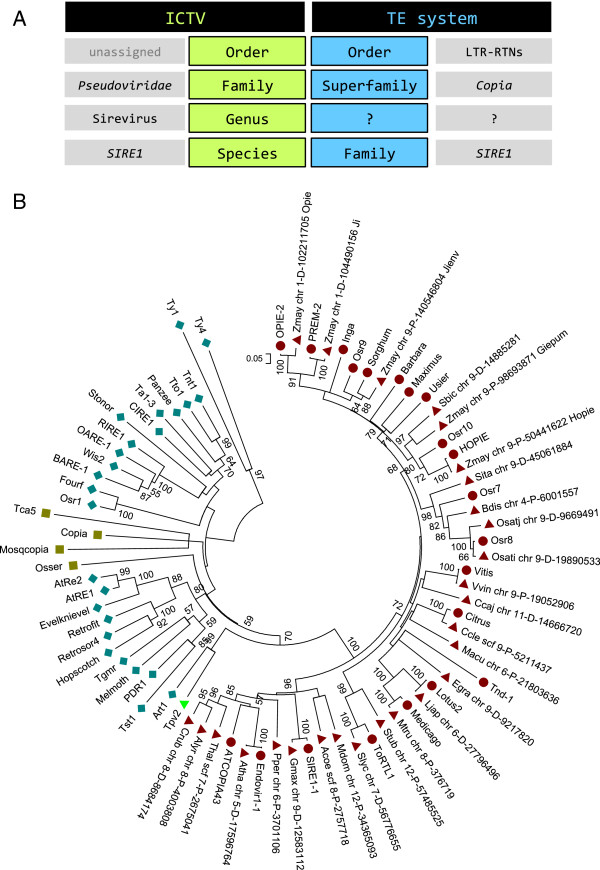
**The *Pseudoviridae*/*Copia *taxonomy and phylogenetic relationships. (A)** The inconsistency in the classification of LTR-RTNs between the ICTV and TE-based systems. **(B)** Neighbor-Joining phylogenetic tree with 1,000 bootstrap replicates based on the sequence of the *pol* polyprotein between the fifth RT conserved domain and the RNase H gene. Sireviruses (red circles, or red triangles if they were retrieved from MASiVEdb) form a monophyletic group, which is supported with 100% confidence by the bootstrap analysis. Pseudoviruses (blue diamonds) and Hemiviruses (brown squares) do not resolve in strongly-supported separate branches. *Tpv2* (green triangle) is a single *Pseudoviridae* species of unknown genera classification.

The confusion is compounded by the discrepancy in the categorization of LTR-RTNs by the two main classification systems, of which only ICTV provides a taxonomic level (‘genera’) between ‘families’ and ‘species’ (or ‘superfamilies’ and ‘families’, respectively, in the TE-based system) (Figure 1A). Each of the three *Pseudoviridae* and *Metaviridae* genera contain a few representative species, which in the majority of cases are known LTR-RTNs. For instance, the name ‘Sirevirus’ was derived from the well-studied *SIRE1* element of the soybean genome [11]. In contrast, the TE-based system is devoid of a similar taxonomic level, resulting in a phylogenetic ‘jump’ from superfamily (for example, *Copia*) to family (for example, *SIRE1*) (Figure 1A).

Perhaps, the reason for this omission was the difficulty in correctly assigning LTR-RTN families into genera. However, in the case of Sireviruses we have shown that both gene-derived phylogenetic analysis and genome characteristics can reliably distinguish Sirevirus elements [19,21,22]. Hence, considering that the TE-based system is the preferred and more suitable scheme for eukaryotic genome studies, this intermediate taxonomic level between superfamilies and families has now become feasible and meaningful.

On the other hand, it is important that LTR-RTNs should remain within the ICTV classification system, even though they are not true viruses. Their evolutionary relationship to retroviruses (*Retroviridae* family) is a long-lasting puzzle that can only be efficiently addressed within the ICTV taxonomy. Aided by the accumulation of new retroviruses and LTR-RTN elements from sequencing projects across the tree of life, it will soon become possible to better understand their phylogenetic pedigree.

### Phylogenetic relationships within the ICTV Pseudoviridae family

The assignment of individual elements into the three *Pseudoviridae* genera is a difficult task, due to significant variances in similarity between the genes of their *gag*-*pol* domain [1]. The initial criterion used in ICTV was the length of the tail of the tRNA molecule that is used as a primer to initiate reverse transcription. Hemiviruses use only a short segment of tRNA in comparison to Pseudoviruses, with no available information for Sireviruses. Neighbor-Joining (NJ) phylogenetic analysis based on the reverse transcriptase (RT) gene was then employed for the between- and within-genus separation into distinct species. As a result, and although the properties of their preferred tRNA molecule were not known, Sireviruses were considered a separate genus, as its representative species were the only ones that consistently featured <45% sequence identity of the RT gene to exemplars of the other two genera [1].

Yet, it was recently shown that, similar to Hemiviruses, Sireviruses use a short 9bp segment of the ^met^tRNA [19]. Furthermore, ICTV only contains a small number of exemplars for each genus, while the incorporation of the vast number of new LTR-RTN species from genome sequencing projects in the classification system is understandably slow or absent. Hence, we decided to reassess the phylogenetic relationships within the *Pseudoviridae* family by conducting a NJ analysis of the downstream section of the *pol* polyprotein defined by the fifth conserved domain of the RT gene [23] to the end of the RNase H gene (Figure 1B). To better capture the diversity of the *Pseudoviridae* family, we used a large set of elements (Additional file 1) including all ICTV exemplars of the three genera, LTR-RTNs used in previous similar studies [15,16,19], and Sirevirus representatives from all plant hosts that are present in MASiVEdb. The phylogenetic tree revealed that Sireviruses form the only monophyletic group (with 100% bootstrap support), whilst there is no clear distinction between Hemiviruses and Pseudoviruses. Our previous analysis also showed that elements of these two genera do not differ in their genome characteristics, in contrast to the unique Sirevirus genome [19].

Consequently, we believe that further research is needed, in part through the addition of more exemplar species in ICTV, to elucidate the phylogenies of Pseudoviruses and Hemiviruses. Moreover, it may be appropriate for ICTV to include the distinctive genome structure of Sireviruses in its phylogenetic-based genus definition. Such criteria may also be used for describing other LTR-RTN genera, if future research uncovers similar findings.

## Conclusions

The ICTV, and especially the TE-based system proposed by Wicker *et al*. [2], are indispensable resources for the challenging identification and annotation of TEs in eukaryotic genomes. However, the explosion of available sequence data and analytical tools strongly support the need for these systems to be revisited and revised: a taxonomic level is now required in the TE-based system that will make the Sirevirus and other ICTV genera available for eukaryotic genome studies. Meanwhile, the phylogenetic relationship of the Pseudovirus and Hemivirus genera should be further clarified. Finally, a similar set of analyses should also take place within the *Metaviridae*/*Gypsy* family/superfamily. Such developments will pave the way for identifying other TE genera with unique characteristics like Sireviruses, and will encourage research that may shed light on their integrative impact on the evolution of their host genomes.

## Competing interests

The authors declare that they have no competing interests.

## Authors’ contribution

AB conceived and coordinated the project, and drafted the manuscript. ND drafted the manuscript. All authors read and approved the final manuscript.

## Supplementary Material

Additional file 1 **List of the RT/RNase H peptide sequences of the elements that were used for the construction of the *Pseudoviridae *phylogenetic tree.** Click here for file
